# Microbiome signatures in neonatal central line associated bloodstream infections

**DOI:** 10.1371/journal.pone.0227967

**Published:** 2020-01-16

**Authors:** Mohan Pammi, Santosh Thapa, Miriam Balderas, Jessica K. Runge, Alamelu Venkatachalam, Ruth Ann Luna

**Affiliations:** 1 Department of Pediatrics, Texas Children's Hospital and Baylor College of Medicine, Houston, Texas, United States of America; 2 Texas Children’s Microbiome Center, Department of Pathology, Texas Children’s Hospital, Houston, Texas, United States of America; 3 Department of Pathology and Immunology, Baylor College of Medicine, Houston, Texas, United States of America; McMaster University, CANADA

## Abstract

Neonates are at high risk for central line associated bloodstream infections (CLABSI). Biofilm formation is universal on indwelling catheters but why some biofilms seed the bloodstream to cause CLABSI is not clearly understood. With the objective to test the hypothesis that catheter biofilm microbiome in neonates with CLABSI differs than those without infection, we prospectively enrolled neonates (n = 30) with infected and uninfected indwelling central catheters. Catheters were collected at the time of removal, along with blood samples and skin swabs at the catheter insertion sites. Microbiomes of catheter biofilms, skin swabs and blood were evaluated by profiling the V4 region of the bacterial 16S rRNA gene using Illumina MiSeq sequencing platform. The microbial DNA load was higher from catheter biofilms of CLABSI patients without differences in alpha diversity when compared to that of the non-CLABSI neonates. *Proteus* and unclassified Staphylococcaceae were more abundant in infected catheter biofilms while *Bradyrhizobium*, *Cloacibacterium*, and *Sphingomonas* were more abundant in the uninfected catheters. A blood microbiome was detected in uninfected samples. The blood microbiome in CLABSI neonates clustered separately from the uninfected blood samples in beta diversity plots. We found that the microbiome signature in catheter biofilm and blood of neonates with CLABSI is different than the microbiomes of non-CLABSI neonates.

## Introduction

Central line-associated bloodstream infections (CLABSI) are a significant component of healthcare-associated infections (HAI) and cause mortality and morbidity [1]. The attributable cost of one specific HAI bloodstream infection ranges from $6000 to $39,000 [2]. Neonates are at higher risk for CLABSI than children or adults, and CLABSI are seen more commonly in neonatal intensive care units (NICU) than in pediatric or adult intensive care units [3]. Higher neonatal risk could be due to the host risk factors, namely breach of physical barriers or relative immunodeficiency, to type of catheters used (silastic percutaneous central line in neonates vs. port-a-cath used in older children and adults), or to contamination during access of the catheters [3]. The incidence of CLABSIs in neonates is 0.9 to 2.5/1000 central line days according to the National Healthcare Safety Network 2011 report [1]. Colonization of the catheter hub and the catheter is the initial event that leads to bloodstream infection [4, 5]. CLABSIs are often caused by organisms colonizing the skin (13%), namely coagulase-negative staphylococci, *Staphylococcus aureus* and *Candida* species [3, 6].

Indwelling catheters are coated by biofilms formed by microorganisms. Biofilms are surface adherent microbial communities, encased in an extracellular matrix, which is composed of polysaccharides, proteins and extracellular DNA [7]. Biofilms are resistant to host defense and antibiotics, difficult to eradicate and require removal of the catheter. Nearly 60–80% of medically important infections are biofilm-related infections [8]. Ultrastructural examination of catheters from patients by scanning electron microscopy demonstrates that biofilm formation is universal and seen as early as 24 hr after catheter placement [9, 10]. Given that biofilm formation is universal and colonization is seen in many catheters, it remains unclear why only some patients develop CLABSI [9, 11].

Molecular microbiological methods such as PCR-based microbial DNA detection may have higher sensitivity to detect biofilm infection [12]. The role of microbial DNA load in neonatal CLABSIs has not been evaluated thus far. To our knowledge, no information exists as to the microbial composition of catheter biofilms or whether changes in the biofilm microbial patterns in terms of diversity or abundance, leads to CLABSIs. Using targeted sequencing of the bacterial 16S ribosomal RNA (rRNA) gene, we evaluated the catheter biofilm microbiome along with skin and blood microbiomes in neonates who had central catheters to determine microbial signatures related to CLABSI progression.

## Materials and methods

### Ethics statements and patient population

This study (H-33004) was approved by the Institutional Review Board at Baylor College of Medicine (Houston, TX). We enrolled neonates with percutaneously inserted central catheters (PICC), excluding umbilical lines, in the NICUs at Texas Children’s Hospital following written informed consent from their parents or guardians.

### Sample collection

Catheters were collected from the patients when they were removed either due to CLABSI or after they were no longer required. CLABSI was defined per the US Centers for Disease Control and Prevention (CDC) criteria (https://www.cdc.gov/nhsn/pdfs/pscmanual/4psc_clabscurrent.pdf). We evaluated 15 PICCs removed from the neonates with CLABSI and 15 PICC lines from neonates without CLABSI. For each of the 30 neonates studied, we also collected skin swabs from the PICC insertion site using a Cytopak, cytosoft brush, CP-5B soaked in sterile 0.15M NaCl with 0.1% Tween 20 (Fisher Scientific, Fair Lawn, NJ) as described in Pammi et al. (2017) [13]. Additionally, 500 μl of circulating blood was collected into EDTA containing tubes from the catheter of each patient just before PICC line removal. Relevant clinical information such as birth weight, and CLABSI status was also recorded (S1 Table). Samples were transported to the laboratory at Texas Children's Hospital on the day of collection. The catheter and skin swab samples were categorized as ‘infected’ if the neonate had CLABSI and otherwise defined as ‘uninfected’. All samples were then transported to the Medical Metagenomics laboratory at the Texas Children’s Microbiome Center for microbiome characterization. Prior to DNA extraction, catheter samples were stored at -80°C while the swabs and blood samples were stored at -20°C.

### DNA extraction

The MoBio Power Biofilm kit (Qiagen, Hilden, Germany) was used for DNA extraction from catheter samples following manufacturer recommendations, with modifications to remove microbes from attached surfaces. Briefly, each catheter was flushed through the hub using 1 mL lysis buffer (1x PBS + 1.2% Triton-X + 2mM EDTA) before being incubated in a shaking heat block (200 rpm) at 37°C for 1 hour. After incubation, each catheter underwent sonication in high power setting using Bransonic® CPX Ultrasonic Bath (Branson Ultrasonics Corp., Danbury, CT) for 5 minutes, and 350 μL of the sonicated lysate was used for DNA extraction.

DNA from skin swabs was extracted using the MoBio PowerSoil DNA Isolation Kit (Qiagen), per manufacturer’s instructions. DNA was extracted from blood samples using the MoBio BiOstic Bacteremia DNA Isolation Kit (Qiagen), with minor modifications as mentioned below. In brief, 500 μL of the blood was centrifuged for 2 minutes at 13,000 x g, with an additional 2 minutes for samples that did not produce visible pellets after initial centrifugation. The resultant DNA from each sample was quantified by a high‐sensitivity dsDNA assay on the Qubit® 2.0 fluorometer (Thermo Fisher Scientific, Inc., Wilmington, DE). The DNA samples were stored at -80°C until further processing.

### 16S rRNA gene qPCR

The bacterial load was quantified using quantitative PCR (qPCR) of the full length 16SRNA gene using a TaqMan® based assay (Applied Biosystems, Foster City, CA). We used the primers and probe set as described previously [14] and reactions were analyzed on a ViiA 7 Real-Time PCR System (Applied Biosystems).

### Microbiome evaluation

The V4 region of the bacterial 16S rRNA gene was amplified (Forward, 5’GACGCTCTTCCGATCTTATGGTAATTGTGTGCCAGCMGCCGCGGTAA3’; Reverse, 5’TGTGCTCTTCCGATCTAGTCAGTCAGCCGGACTACHVGGGTWTCTAAT3’) using the NETFlex® V4 Amplicon-Seq Kit 2.0 (Bio Scientific, Austin, TX) with 20 ng of input DNA as described in Luna et al. (2017) [15]. The library concentration was quantified by measuring the PCR amplified product of each sample using a high-sensitivity dsDNA assay on the Qubit (Thermo Fisher Scientific, Inc.). Purified 16S libraries were pooled in equimolar amounts to a final concentration of 7 pM. Paired-end sequencing (2x250) was performed on an Illumina MiSeq® instrument (Illumina, San Diego, CA) at the Texas Children’s Microbiome Center. Raw sequence data were processed with the LotuS pipeline (v1.462) as previously described [16]. In brief, raw reads were demultiplexed and quality filtered due to sequence length (read 1: min/max = = 170/250bp), quality (avg quality score = 27, avg quality score over a window of 50 nucleotides = 25), homopolymer length (max = 8), ambiguous bases (max = 0), barcode and primer errors (max = 0), and accumulated error over the sequence. Filtered sequences were then grouped into operational taxonomic units (OTUs) at 97% identity by a closed-reference approach against the SILVA database (v123) using the UPARSE (USEARCH v8.0.1623) clustering algorithm [17]. Chimeric sequences were removed using vsearch (v1.9.7) [18]. Taxonomic assignments were performed using the RDP classifier (v2.2) by comparison against the SILVA database (v123) [19] at 80% confidence. OTUs failing to classify as bacteria at the kingdom level and unclassified OTUs at the phylum level were removed prior to further analysis.

The microbiome composition and diversity was estimated only in samples with greater than 100 quality-filtered reads/sample [20]. Relative abundances of bacteria at various taxonomic levels were calculated in each sample type for both the uninfected and infected cohort. Alpha and beta diversity metrics were generated using the open-source software QIIME (v1.9.1) [21]. Bacterial alpha diversity was estimated with data rarefied to the lowest sequencing depths for each sample type. Observed OTUs and the Shannon’s diversity indices were calculated. Principal coordinates analysis (PCoA) plots of the unweighted and weighted UniFrac distances were used to assess beta diversity between the samples. Bacterial composition and diversity results were compared among groups based on the available clinical information.

### Statistical analysis

The statistical analysis of metagenomic profiles (STAMP) (v2.1.3) was used to analyze the bacterial community profiles [22]. Nonparametric tests were applied because our data did not meet normality assumptions using the Shapiro-Wilk test. Comparisons among multiple groups utilized a Kruskal-Wallis test, while the two-sided Mann-Whitney test was used for comparing two groups. For significant Kruskal-Wallis tests, a post-hoc analysis was performed to determine which levels of the independent variable differ from each other level using the Dunn test. When performing multiple comparison testing, adjustments to the p-values were made using the Benjamini-Hochberg correction to control the false-discovery rate (FDR) at 0.05 [23]. Comparisons of UniFrac distance matrices among and within groups were performed using a permutational multivariate analysis of variance (PERMANOVA) in the vegan R-package (v2.5.3) using Phyloseq (v1.24.2).

### Distinguishing the low biomass microbiome from possible background contaminants

To monitor the background contamination, we prepared several types of negative controls for each set of DNA extraction in parallel with the samples. These included (a) empty tubes that were processed through DNA extraction without adding the extraction reagents (“kit control”), (b) tubes of molecular biology grade water used for DNA elution (“water control”) and (c) tubes of the lysis buffer used for DNA extraction from catheter samples (“buffer control”). The bacterial load in our samples were compared with that of the negative controls to determine whether the DNA extracted from the samples contained higher levels of bacterial DNA than negative controls. In addition, a total of 10 negative controls (4 buffer controls and 3 each for the kit and water controls) prepared during the experiments were processed for sequencing along with the samples to monitor possible background contaminants.

We also applied a recently published *decontam* R-package (v1.2.1) [24] to our dataset (in samples with >100 quality-filtered reads/sample) to further identify and visualize any contaminating OTUs. Phyloseq R-package was used to generate input objects for *decontam* analysis. We inspected the frequencies of OTUs as a function of DNA concentration (range: undetectable to 100 ng/μL) measured by the Qubit assay after 16S V4 PCR and prior to Miseq sequencing. An OTU is classified as a contaminant or non-contaminant by comparing its associated score statistic *P* (*decontam* score) to the default classification threshold of 0.1.

### Data availability

All 16S rRNA raw sequence data generated during this study has been deposited in the National Center for Biotechnology Information (NCBI) sequence read archive (SRA) under the BioProject ID- PRJNA596209.

## Results

### Patient characteristics

The neonates in the non-infected group had a mean birth weight of 1774 g, gestational age of 32 weeks, and a mean of 20 catheter days. The infected group (with CLABSI) had a mean birth weight of 1454 g, gestational age of 30 weeks, and a mean of 24 catheter days. All but one neonate received antibiotics treatment, with a mean duration of 8 days for non-CLABSI and 11 days for CLABSI groups. Ampicillin and Gentamicin were administered either alone or in combination with other antibiotics in 97 and 93% of the total subjects, respectively (see Tables 1 and S1).

**Table 1:** Cohort summary.

Characteristics	Non-CLABSI neonates (n = 15)	CLABSI neonates (n = 15)	*p*-value ^a^
*Gestational age (weeks)*			
Mean (± SD)	32 (± 6)	30 (± 5)	0.42
*Birth weight (gram)*			
Mean (± SD)	1774 (± 968)	1454 (± 973)	0.26
*Catheter days*			
Mean (± SD)	20 (± 17)	24 (± 21)	0.95
*Number of NEC cases*	1/15 (6.7%)	5/15 (33.3%)	–
*Maximum feeds (ml/kg/day)*			
Mean (± SD)	122 (± 34)	48 (± 40)	< 0.0001
*Predominant nutrition (>50%)*			
TPN:Enteral feeds ^**b**^ (%)	40:60	100:0	0.0007
*Ethnicity*			
Hispanic:Non-Hispanic (%)	20:80	40:60	0.43
*Antibiotics use*			
Antibiotics course (mean days ±SD)	8 ± 9	11 ± 7	0.05
Subjects administered antibiotics (n)	14	15	–
Antibiotics [n (% of total subjects)] ^**c**^			
Gentamicin	13 (93%)	15 (100%)	–
Ampicillin	14 (100%)	13 (87%)	–
Vancomycin	7 (50%)	15 (100%)	–
3^rd^ generation Cephalosporins	5 (36%)	10 (67%)	–
Clindamycin	5 (36%)	11(73%)	–

CLABSI, Central line associated bloodstream infections; NEC, Necrotizing enterocolitis; TPN, Total parenteral nutrition.

^**a**^*p* values were calculated using the Mann-Whitney test, except the proportions of TPN:Enteral feeds and Hispanic:Non-Hispanic in non-CLABSI and CLABSI neonates (Fisher's exact test). A *p* value of <0.05 is considered significant.

^**b**^Enteral feeds includes both mother’s expressed breast milk (MEBM) and donor expressed breast milk (DEBM).

^**c**^Selected category of antibiotics are shown.

### Blood culture isolates

The most common organism isolated by blood culture within 3 days of catheter removal was coagulase-negative staphylococci (CONS) (9/30, 30%), followed by *Staphylococcus aureus* (4/30, 13.3%). *S*. *epidermidis* was the major CONS (5/9, 55.5%) isolated from blood culture (S1 Table for details).

### 16S V4 rRNA sequencing output

Table 2 provides a summary of the samples processed and analyzed for the study. A summary of sequencing output, including number of reads, quality metrics, and number of OTUs, is presented in Table 3. The raw data generated from our samples (n = 90) had an average of 32,539 reads/sample with ≥Q30 quality score of 64% (see Table 3). However, a total of 7,516 reads (read range: 1–354) belonging to 187 different OTUs (OTU abundance range: 1–2,291) assigned as ‘kingdom_bacteria’ were characterized as ‘unclassified_bacteria’ at the phylum level using the custom pipeline described above.

**Table 2:** Summary of samples processed and analyzed in this study.

Sample type	Infection status^a^	Number of samples processed initially	Final number of samples analyzed^b^ (after removal of unclassified OTUs at kingdom & phylum levels)
Catheter biofilm	Uninfected	15	12
	Infected	15	15
Skin Swab	Uninfected	15	5
	Infected	15	6
Blood	Uninfected	15	3
	Infected	15	3
**Total samples**		**90**	**44**

^a^ CLABSI was determined based on the CDC guidelines as described in the Methods. The catheter and skin swab samples were categorized as ‘infected’ if the neonate had CLABSI and otherwise defined as ‘uninfected’.

^b^ Samples were included in the final analysis only if these yielded usable amount of sequencing reads (>100 quality-filtered reads per sample).

**Table 3:** Summary of sequencing depth, quality and number of OTUs identified in the samples.

Features	Raw reads (90 samples)	Quality-filtered* reads with >100 reads/ sample (44 samples)-before decontam	Quality-filtered reads with >100 reads/ sample (44 samples)-after decontam
*Number of Reads*			
Minimum:	175	104	96
Maximum:	73,107	45,189	45,180
Mean:	32,539	9,227	9,122
Total:	2,928,499	406,007	401,381
*Quality (mean)*			
% PF clusters	54	——	——
% ≥ Q30 score	64	——	——
Mean quality score	30	——	——
*Total OTUs*	——	440^#^	412^##^

* after removing chimera, and unclassified OTUs at kingdom and phylum levels using SILVA (v123) database.

^*#*^ including 18 different singleton OTUs.

^*##*^ including 16 different singleton OTUs.

Unexpectedly, all OTUs assigned as ‘kingdom_bacteria; phylum_unclassified_bacteria’ were found to be non-bacterial reads, and almost all of them aligned to human DNA using the NCBI nr/nt and the human genomic plus transcript databases. We thus removed the OTUs failing to classify as bacteria at the kingdom level and unclassified OTUs at the phylum level from downstream analysis. After removing unclassified OTUs at both the kingdom and phylum levels, only 44 (out of 90) samples yielded a useable amount of quality-filtered sequencing data (>100 reads/sample), totaling 406,007 reads belonging to 440 different OTUs.

### Application of *decontam* R-package

A total of 28 OTUs were identified as contaminants by the frequency based *decontam* classification at a default threshold (0.1) (see S2 Table and S1 Fig for details). Application of the *decontam* statistical method to our samples (n = 44, each with >100 reads/sample) resulted in an average of 9,122 reads/sample (range: 96–45,180) assigned to 412 different OTUs (Table 3). Downstream analysis, including bacterial diversity and composition was assessed in the contaminants removed dataset.

### Bacterial load

**Fig 1:**
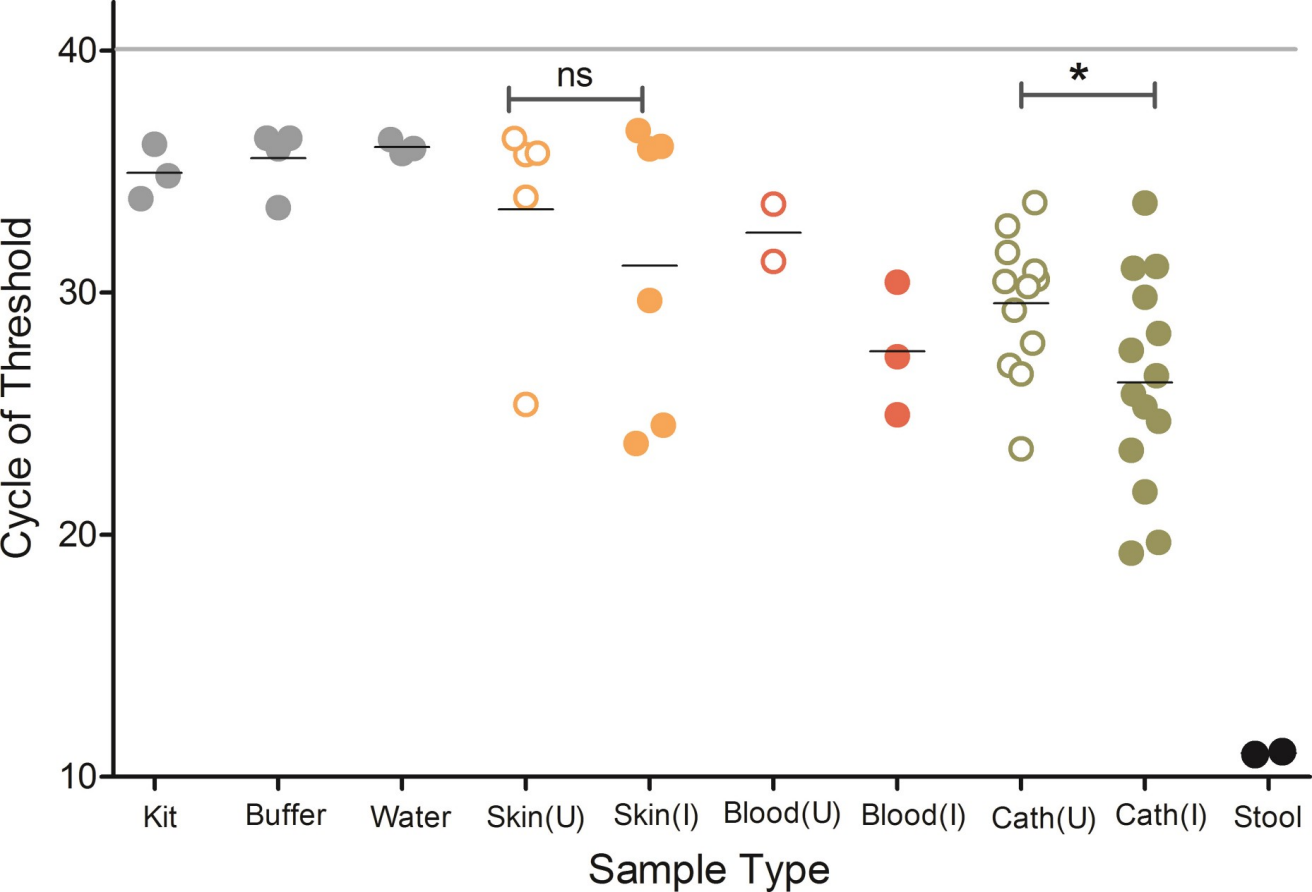
The bacterial load is higher in biofilms of infected catheters. qPCR analysis of the bacterial 16S rRNA gene abundance in various samples studied. Significantly higher levels of bacterial DNA was detected in the infected catheters (n = 14) compared to the uninfected catheters (n = 12) (Mann-Whitney test p<0.05). *p<0.05, ns = non-significant (p>0.05). Cath = catheter, U = uninfected, I = infected.

Samples from infected catheters (n = 14) contained a significantly higher bacterial load (low cycle of threshold (CT)) than the uninfected catheters (n = 12) as measured using 16S rRNA gene qPCR (Mann-Whitney test p<0.05) (Fig 1). All negative controls (leftmost three sample sets in Fig 1) showed high CT values, indicating low content of 16S rRNA gene. In contrast, stool samples used as positive controls had a high concentration of bacterial DNA (low CT), as expected (rightmost sample set in Fig 1). The bacterial load was also significantly higher in both infected and uninfected catheters than the water and buffer controls (Kruskal Wallis with Dunn’s post-hoc test p<0.05). No significant difference was found in the bacterial load between the infected and uninfected skin swabs (Mann-Whitney test p>0.05). There was no significant difference between skin swab samples and the negative controls either (Kruskal Wallis with Dunn’s post-hoc test p>0.05). We did not perform statistical comparison of the bacterial load between infected and uninfected blood samples because of the small sample size. The 16S qPCR result was similar to that of the Qubit result (measured using PCR amplified 16S V4 rRNA gene) (see S2 Fig).

### Alpha diversity of the microbiome from the catheter, blood and skin swab

**Fig 2:**
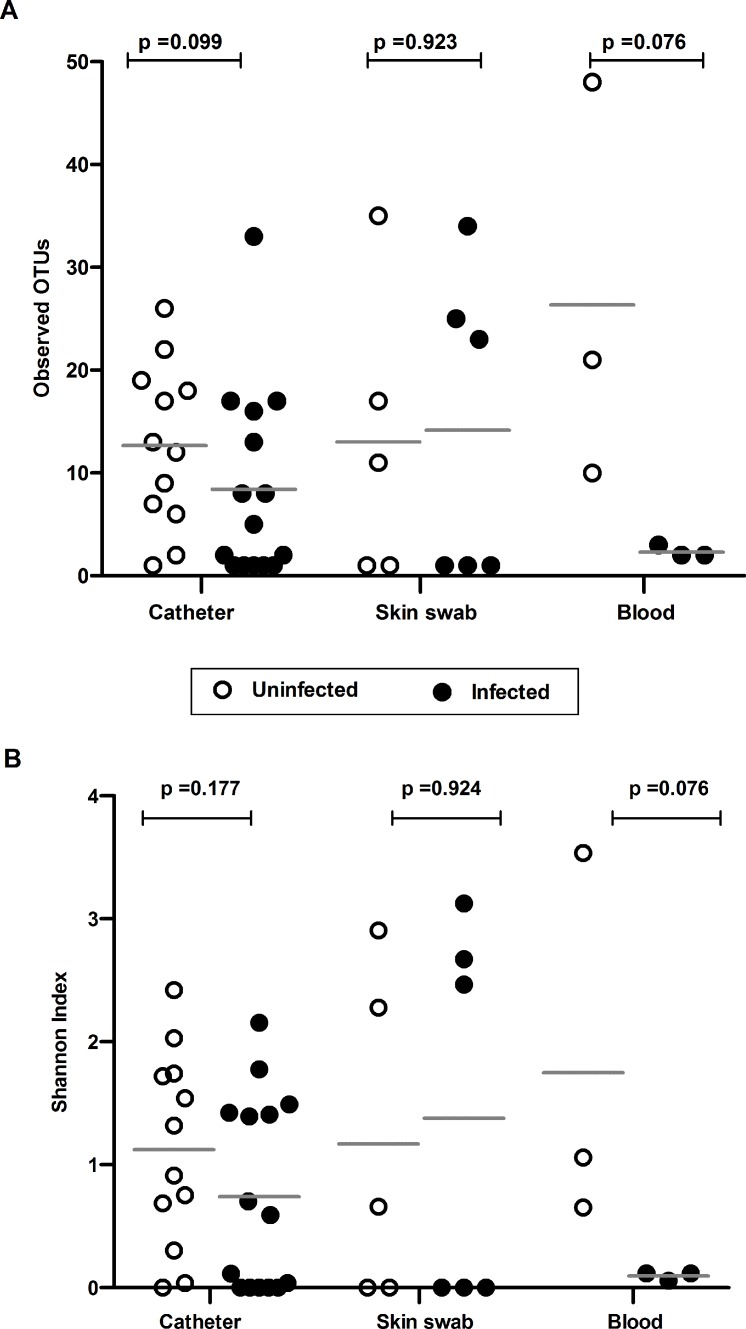
Alpha diversity metrics in CLABSI and non-CLABSI neonates. (**A**) Observed OTUs and (**B**) the Shannon Diversity Indices are presented in scatter plots. The alpha diversity metrics did not differ between infected and uninfected catheters.

Alpha diversity within the specimens was measured in the contaminant-removed dataset, rarefied to an even sampling depth (catheter = 165 sequences, blood = 137 and skin swab = 96). There was no statistically significant difference (Mann-Whitney test p>0.05) in the microbial richness (as measured by observed OTUs, Fig 2A) or diversity (as estimated by the Shannon diversity index (SDI), Fig 2B) based on CLABSI status for catheter samples. We also found no differences in the richness and diversity between uninfected and infected groups in skin swab and blood samples. Similar results were obtained with the non-rarefied dataset (S3 Fig).

### Beta diversity of samples from infected (CLABSI) and uninfected neonates

**Fig 3:**
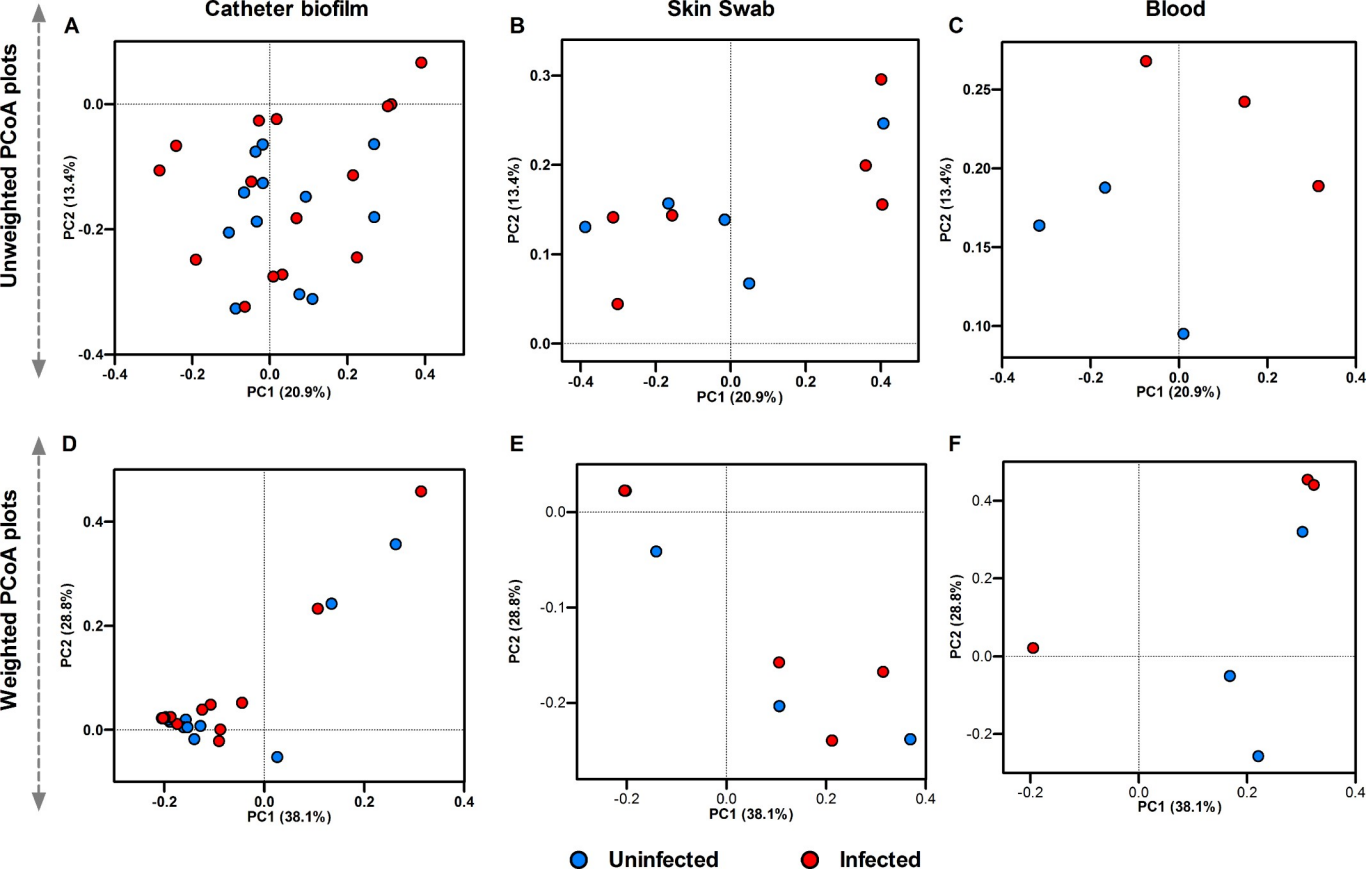
Multivariate analysis of beta diversity. PCoA plots of the microbial communities in infected (red circles) and uninfected samples (blue circles) of (**A**) catheter biofilms, (**B**) skin swab and (**C**) blood respectively as measured using unweighted UniFrac distances. Uninfected blood samples are clustered separately from the infected blood samples (see Fig 3C), but there is no clustering identified in the catheter biofilm or skin swab microbial communities. Figs **D**-**F** represents scatter plots of the weighted UniFrac distance metrics for the catheter, skin swab and blood microbiomes in uninfected and infected groups. Each circle represents the complete microbial community of a biological sample. The first 2 principal components (PC1and PC2), along with the amount of variation explained are shown in the figures.

Multivariate analyses of beta diversity measured by UniFrac distance matrices are presented in principal coordinate analysis (PCoA) plots (Fig 3A–3F). We did not observe significant clustering by the infection status for any of the sample types using unweighted UniFrac distance matrices (Fig 3A–3C) (uninfected vs infected groups: PERMANOVA p>0.05 for all sample types). Similar results were obtained with weighted UniFrac distances (Fig 3D–3F). However, the blood microbiomes of infected subjects clustered separately from the uninfected subjects (see Fig 3C).

### Microbial composition and structure

#### Microbiome composition by specimen type

The microbial community composition differed by sample type. Based on specimen type, we found a higher relative abundance of the phylum Firmicutes in both catheter biofilm (BH corrected Dunn’s Kruskal-Wallis test p = 0.03) and skin swab (p = 0.02) when compared to the abundance in blood (S4 Fig). One of the major CLABSI bacteria- *Staphylococcus* spp. was found at a significantly higher abundance in catheters (mean relative abundance = 63%) and skin swabs (59%) than in blood (16%) samples (p<0.05 for both comparisons, S4 and S5 Figs). Additionally, the mean abundance of *Streptococcus* was significantly higher in catheters (6%) compared to the mean abundance in skin swabs (0.01%) (p = 0.0001). Other genera belonging to Firmicutes, such as *Enterococcus* and *Anaerococcus* were only present in catheter biofilm samples (see S4 and S5 Figs).

#### Catheter microbiomes of CLABSI and non-CLABSI neonates

**Fig 4:**
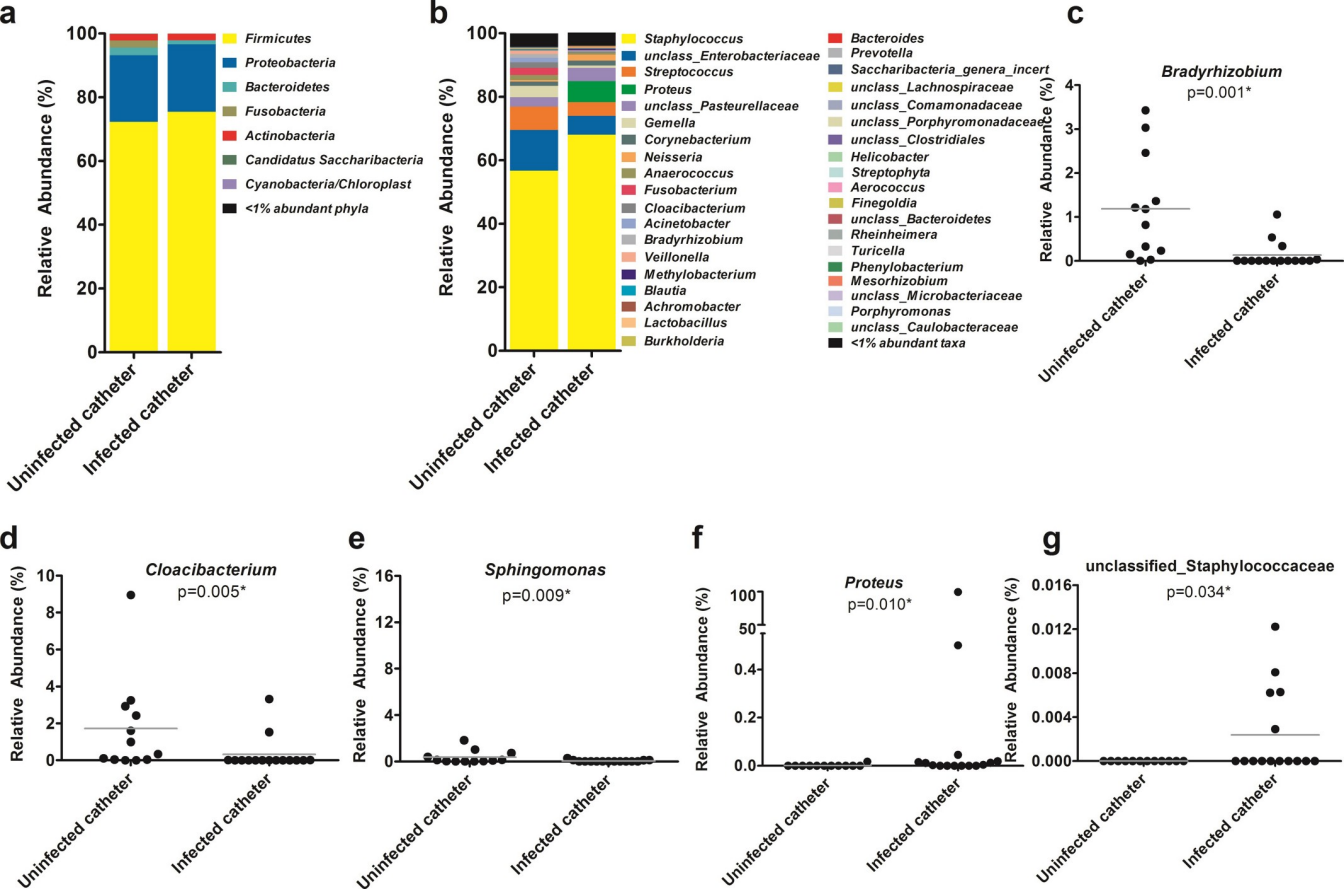
The microbiome profile of infected catheters is distinct from uninfected catheters. Bar plots representing the taxonomic composition of the catheter biofilm microbiota at the (**A**) phylum and (**B**) genus level for uninfected (n = 12) and infected (n = 15) catheters. Taxa with a mean relative abundance <1% are grouped together. Comparisons between infected and uninfected groups used a Mann-Whitney test. There was a significantly (p<0.05) lower abundance of *Bradyrhizobium*, *Cloacibacterium*, and *Sphingomonas* in infected catheters when compared to uninfected catheters (**C**-**E**). In contrast, infected catheter samples had a higher proportion of (**F**) *Proteus* and (**G)** unclassified Staphylococcaceae in comparison to the uninfected catheters.

We found no statistically significant differences in relative abundance of the bacterial phyla between infected and uninfected catheters (Mann-Whitney test p>0.05, Fig 4A). However, a group of bacteria significantly differ in their relative abundances between infected and uninfected catheters (p<0.05, see S3 Table and Fig 4B for details) at the genus level. Among the genera with ≥1% mean relative abundances across the group, we found a significantly lower abundance of *Bradyrhizobium* (p = 0.001) and *Cloacibacterium* (p = 0.005) in infected catheters when compared to the uninfected catheters (Fig 4C and 4D, respectively). In contrast, the relative abundance of *Proteus* was significantly higher (p = 0.01) in infected catheters than the non-infected catheters (Fig 4F). In the case of taxa with <1% relative abundances, Staphylococcaceae_unclassified (assigned to a single OTU-337) had a higher abundance (p = 0.034) in the infected catheters compared to the uninfected catheters (Fig 4G), among others (S3 Table).

*Staphylococcus*, the predominant bacteria in skin samples, was found with a relative abundance of more than 50% in both infected and non-infected catheter microbiomes.

#### Skin swab microbiomes in CLABSI and non-CLABSI neonates

**Fig 5:**
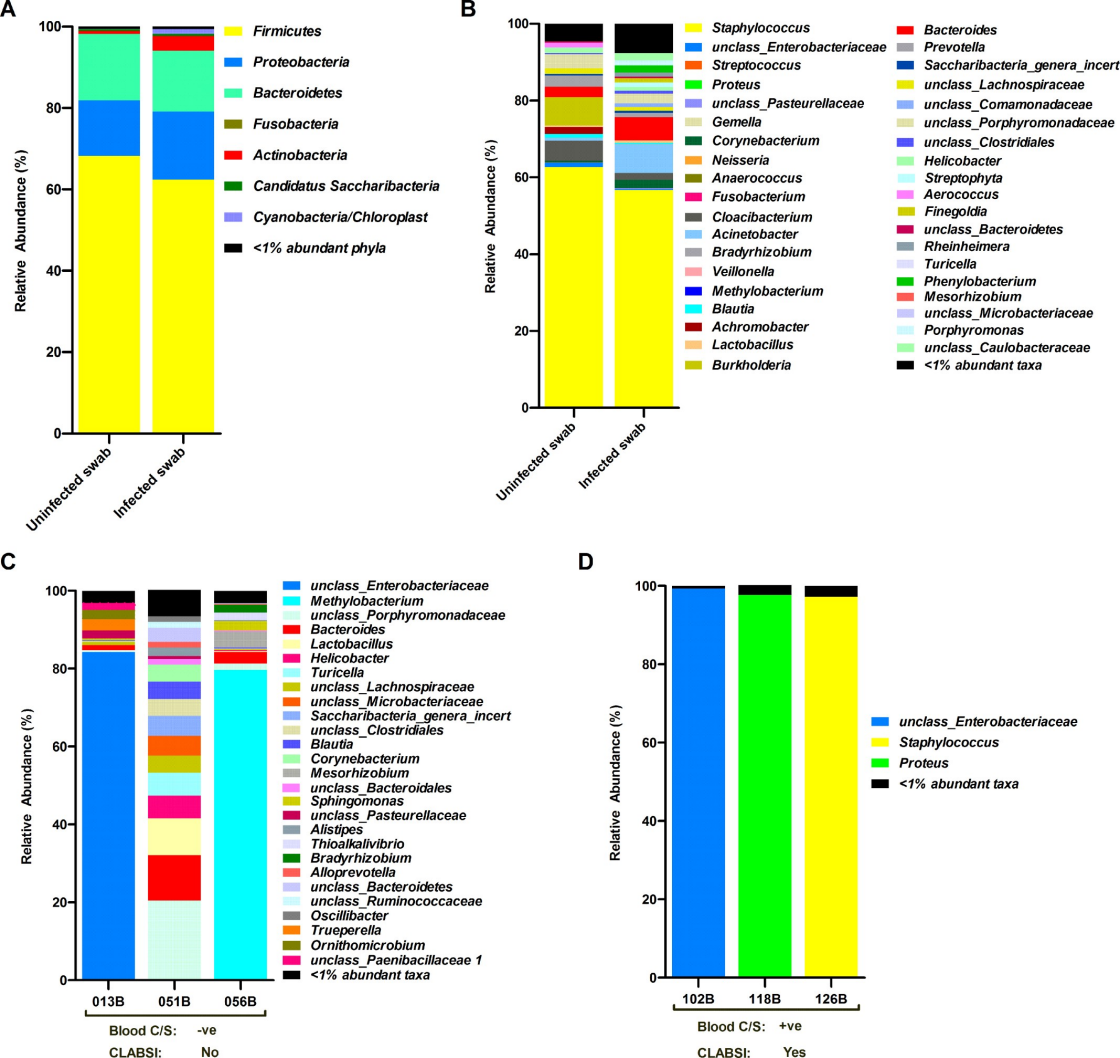
Skin and blood microbiome composition of CLABSI and non-CLABSI neonates. Columns represent the average relative abundance of bacterial taxa at (**A**) phylum and (**B**) genus level for uninfected (n = 5) and infected (n = 6) skin swabs collected from the non-CLABSI and CLABSI neonates, respectively. Bar plots showing the relative abundances of bacteria in individual blood samples collected from (**C**) blood culture negative (non-CLABSI) and (**D**) blood culture positive (CLABSI) neonates (identified on the x-axis). The results of the blood microbiomes are not combined for uninfected and infected groups because each individual within the group are very different in terms of their blood microbiome composition.

Skin swab microbiomes of CLABSI neonates did not differ significantly from that of the non-CLABSI individuals (Mann-Whitney test p>0.05) both at the phylum and genus level (Fig 5A and 5B), with the exception of a very rarely abundant family level taxa- unclassified Oxalobacteraceae.

#### Microbiomes in blood samples of CLABSI and non-CLABSI neonates

Among the six blood samples (collected from six different individuals) included in the final analysis of the blood microbiomes, three samples (013B, 051B and 056B) were ‘culture-negative’ by traditional blood culture methods and reported as uninfected (i.e. non-CLABSI). However, 16SV4 rRNA sequencing of these non-CLABSI blood samples revealed the presence of many bacterial taxa, including *Bacteroides*, *Clostridiales*, *Helicobacter*, and unclassified Enterobacteriaceae (Fig 5C). On the other hand, 16S sequencing of the ‘culture-positive’ blood samples (n = 3) revealed all the bacterial genera identified by traditional culture-based methods (Fig 5D).

#### Catheter microbiomes by type of nutrition

When comparing the two predominant nutritional groups of TPN and enteral feeds (MEBM and DEBM combined), we found multiple bacterial taxa with significantly different relative abundances (Mann-Whitney test p<0.05) in the catheter microbiomes between the groups (S4 Table). However, both alpha and beta diversity of the catheter microbiomes between the groups was not different (Mann-Whitney test of Shannon index p>0.05, unweighted UniFrac PERMANOVA p> 0.05).

## Discussion and conclusions

We detected differences in the microbiome signatures in catheters of neonates with CLABSI in comparison to that of the microbiomes from non-CLABSI neonates. We found that the bacterial load (measured using 16S qPCR) from biofilms of infected catheters (CLABSI neonates) was significantly greater than that of the uninfected catheters (non-CLABSI neonates), but this difference was not seen in the skin swabs collected at the site of catheter insertion. Although statistical comparison of the bacterial load between uninfected blood and infected blood was not possible because of the very small sample size, infected blood samples generally had a higher bacterial load (low CT) than the uninfected blood specimens. Our findings of a higher amount of ‘contaminant’ sequences (i.e. the OTUs assigned as ‘kingdom_bacteria; phylum_unclassified_bacteria’) in the skin swab and blood samples (blood and skin together constituted 6,130 reads out of 7,516 total contaminant reads) may explain the nonsignificant differences. Almost all of the contaminant OTUs matched to the *Homo sapiens* reference genome rather than any bacterial taxa, suggesting non-specific PCR amplification of human DNA in our samples. In addition to removing the human-derived OTUs (discussed above), application of a newly described *decontam* R-package to our data helped further identify, visualize and remove contaminating OTUs, thereby providing a more accurate picture of the sampled microbial communities.

Studies have shown that neonatal sepsis is often associated with lower colony forming units of bacteria in the blood [25, 26], which may contribute to the low biomass of the extracted microbial DNA. A previous study demonstrated that the microbial DNA load of blood (> 0.5 pg/μl) collected from the catheters (n = 207) in children with cancer can detect catheter infections in 61% (95% CI 44 to 83%) of those classified as probable central venous catheter-associated infection with 88% specificity (95% CI 84 to 92%) [27]. High DNA load was associated with higher specificity for diagnosing catheter infection and predicted subsequent catheter removal in this study. However, to our knowledge, there are no neonatal studies that have evaluated and measured the catheter biofilm DNA. Early monitoring of the microbial DNA load in catheter biofilms may be useful in predicting and possibly preventing catheter associated infections. Molecular tools and techniques would further enable us to detect and discriminate very low microbial biomass infections.

Catheter tip cultures have been used to confirm catheter infection after removal, but culture methods may be insensitive for identifying the organisms in the catheter biofilm [28]. Antibiotic exposure may further decrease the sensitivity of microbial culture methods. Indwelling catheters are universally coated with biofilms that are often polymicrobial, and the reason why some progress to CLABSI is not known [9, 11]. One possible explanation is that when the microbial DNA load of these biofilms exceeds a certain threshold, biofilms disperse and seed the bloodstream as CLABSI. The diversity and composition of the microbial communities within in the biofilms may contribute to biofilm dispersal and CLABSI.

Our findings of a high abundance of many genera belonging to the phylum Firmicutes, including *Staphylococcus* spp., in catheter samples supports previous work in this area. *Staphylococcus* spp. has been shown to form extensive biofilms on plastic devices [29], so it is likely that the inherent ability of this organism to form biofilms may have resulted in a high abundance in catheter samples.

We found significantly higher relative abundances of multiple bacterial genera, including *Bradyrhizobium*, *Cloacibacterium*, and *Sphingomonas*, in the catheter microbiomes of the neonates with non-CLABSI than the CLABSI cohorts. However, the significance of the presence of bacteria, including *Bradyrhizobium*, in non-infected catheter is not clear; *Bradyrhizobium* spp. are gram-negative bacilli found in the soil and are responsible for nitrogen fixation. In contrast, higher abundance of bacteria such as *Proteus* and Staphylococcaceae_unclassified in the infected catheters than the uninfected catheters suggests possible roles of these bacteria during CLABSI infection. Using a mouse model of catheter biofilm infection, we have previously demonstrated that an increased extracellular DNA of *Staphylococcus epidermidis* in polymicrobial biofilm is associated with greater systemic dissemination of the bacterium [29]. It has also been shown that biofilm formation may be enhanced by parenteral nutrition, which may also increase bacterial colonization [30]. In fact, all patients in our CLABSI cohort were on total parenteral nutrition, suggesting that TPN may have contributed to the higher abundance of biofilm producing bacteria such as Staphylococcaceae_unclassified, *Proteus* spp. in the infected catheters. Surprisingly, we detected no differences in the relative abundance of Staphylococcaceae_unclassified, but *Proteus*, between neonates receiving TPN (n = 20) and those receiving enteral feeds (n = 7, MEBM and DEBM combined), indicating that the impact of TPN on the abundance of members of the Staphylococcaceae family may depend on the infection status (CLABSI or not) of the neonates.

We evaluated the microbiome of the skin at the insertion site of the catheter because skin is often the portal of entry of organisms into a catheter. We did not find major differences in the skin microbiome around the catheter entry when infected (CLABSI) or not. The skin swab microbial profiles observed in the present study, such as dominance of the phylum Firmicutes (74%), lies in agreement with our previous study on the longitudinal development of cutaneous microbiota of the skin across multiple sites (forehead, antecubital fossa and the gluteal region) during the neonatal period [13].

An interesting finding in this study is that the blood of neonates whose blood cultures were negative and who were considered non-infected (i.e. blood culture-negative non-CLABSI neonates), contained microbial DNA and had a microbial profiles comprised of many bacterial taxa, including *Bacteroides*, *Blautia*, *Clostridiales*, *Helicobacter*, and *Methylobacterium*. The higher abundance of bacterial genera such as *Staphylococcus* and *Proteus* in the blood samples of CLABSI neonates, compared to the neonates without CLABSI, indicates a potentially pathogenic bacterial species. Despite being diagnosed with a blood stream infection by traditional culture-based methods, we were unable to recover useable amount of reads by 16S sequencing for 12 of the CLABSI patients, due to non-specific PCR amplification. Therefore, in light of the small number of blood samples that remained for analysis after quality control (n = 3, each for CLABSI and non-CLABSI groups), caution is warranted in interpretations of these differences. The clinical implications of having microbial DNA in healthy neonates are not known. A circulating blood microbiome in healthy and disease states has been demonstrated, and the assumption that blood in healthy humans is sterile has been questioned [31–36]. The source for the blood microbial DNA is not clear although translocation across the gut epithelium may be the major route and others, such as the oral cavity, skin, lung and vagina, may also contribute [32]. Translocation from the gastrointestinal tract may involve bacteria, bacterial components and products such as lipopolysaccharide and teichoic acid, and viruses [37–40]. Neonatal sepsis after 3 days of life has been found to be originated often from the gut and the skin and a recent study by Stewart et al (2017) confirmed the association of gut bacteria and neonatal sepsis [41]. In our cohort, none of the blood samples were from neonates with NEC, but the physiological immaturity of the gut barrier in preterm infants may have contributed.

Investigators have long argued that healthcare-associated infections, especially CLABSIs, may be considered medical errors and essentially preventable [42]. NICU-wide efforts in catheter care bundles and policies, quality improvement efforts and collaboration on a nation-wide basis is strongly encouraged for reduction in CLABSIs [42–46]. Evaluation of catheter biofilm microbial signatures may help us elucidate the pathophysiology of CLABSI. Detecting a bacterial load is a novel strategy that may permit earlier diagnosis of CLABSIs. Earlier detection may enable earlier targeted therapy, such as antimicrobial lock solutions and may facilitate preservation of catheters in this vulnerable population. Catheter microbial DNA signatures may be useful biomarkers to predict or diagnose infections and may also serve as a monitoring tool to confirm resolution of infection.

## Supporting information

S1 Fig Contaminants OTUs identified using *decontam* statistical package. The frequencies of OTUs were inspected as a function of DNA concentration (range: 0.087 to 100 ng/μL) measured by Qubit assay after 16S V4 PCR and prior to Miseq sequencing. An OTU is classified as contaminant or non-contaminant by comparing its associated score statistic *P (decontam score)* to the default classification threshold of 0.1. A total of 28 OTUs were identified as contaminants using the frequency based *decontam* classification at the default threshold. The frequency of all contaminant OTUs showed negative correlation with the total DNA concentration (red line). The horizontal black dot line represents the expected frequency of non-contaminant OTUs.
(PDF)

S2 Fig PCR amplified 16S V4 rRNA gene concentration in various samples measured by high sensitivity dsDNA Qubit assay. Significantly higher amount of the PCR amplicon was detected in the infected catheters (n = 15) compared to the uninfected catheters (n = 12) (Mann-Whitney test p<0.05). There was no significant difference in the amount of 16S V4 PCR amplicon between infected and uninfected skin swabs (p>0.05) as well as between the skin samples and the negative controls (Kruskal Wallis with Dunn’s post-hoc test p>0.05).
(PDF)

S3 Fig Alpha diversity metrics in CLABSI and non-CLABSI neonates using non-rarefied dataset. (**A**) Observed OTUs and (**B**) the Shannon Diversity Indices are presented in scatter plots.
(TIF)

S4 Fig Bacterial communities by specimen type. Bar plots show (**A**) phylum and (**B**) genus (wherever possible) level bacterial taxa present in the catheter biofilm (n = 27), skin swab (n = 11) and blood (n = 6) samples of the uninfected and infected neonates combined together. Taxa at ≥1% relative abundance in at least one of the samples in each group are reported individually, while those with <1% abundance are grouped together under ‘<1% abundant’ category.
(TIF)

S5 Fig Clinically important and differently abundant bacterial taxa in catheter biofilm, skin swab and blood samples. Catheter biofilm, n = 27; skin swab, n = 11 and blood, n = 6. The p-values mentioned in the graphs (**A**-**E**) were obtained using Kruskal-Wallis test followed by Dunn’s multiple comparisons and adjusted to control false discovery rate using Benjamini-Hochberg correction.
(TIF)

S1 Table Clinical information of the patients under study. (DOCX)

S2 Table Number of reads in decontam-identified contaminant OTUs. (DOCX)

S3 Table Differently abundant bacterial taxa in uninfected and infected catheter biofilm samples. (DOCX)

S4 Table The effect of nutrition on catheter microbiota composition in neonates. (DOCX)
