# Supplementary figures and images for: Microbiome signatures in neonatal central line associated bloodstream infections
Source: PLoS One. 2020 Jan 16;15(1):e0227967. doi: 10.1371/journal.pone.0227967 (PMC6964844; doi:10.1371/journal.pone.0227967)

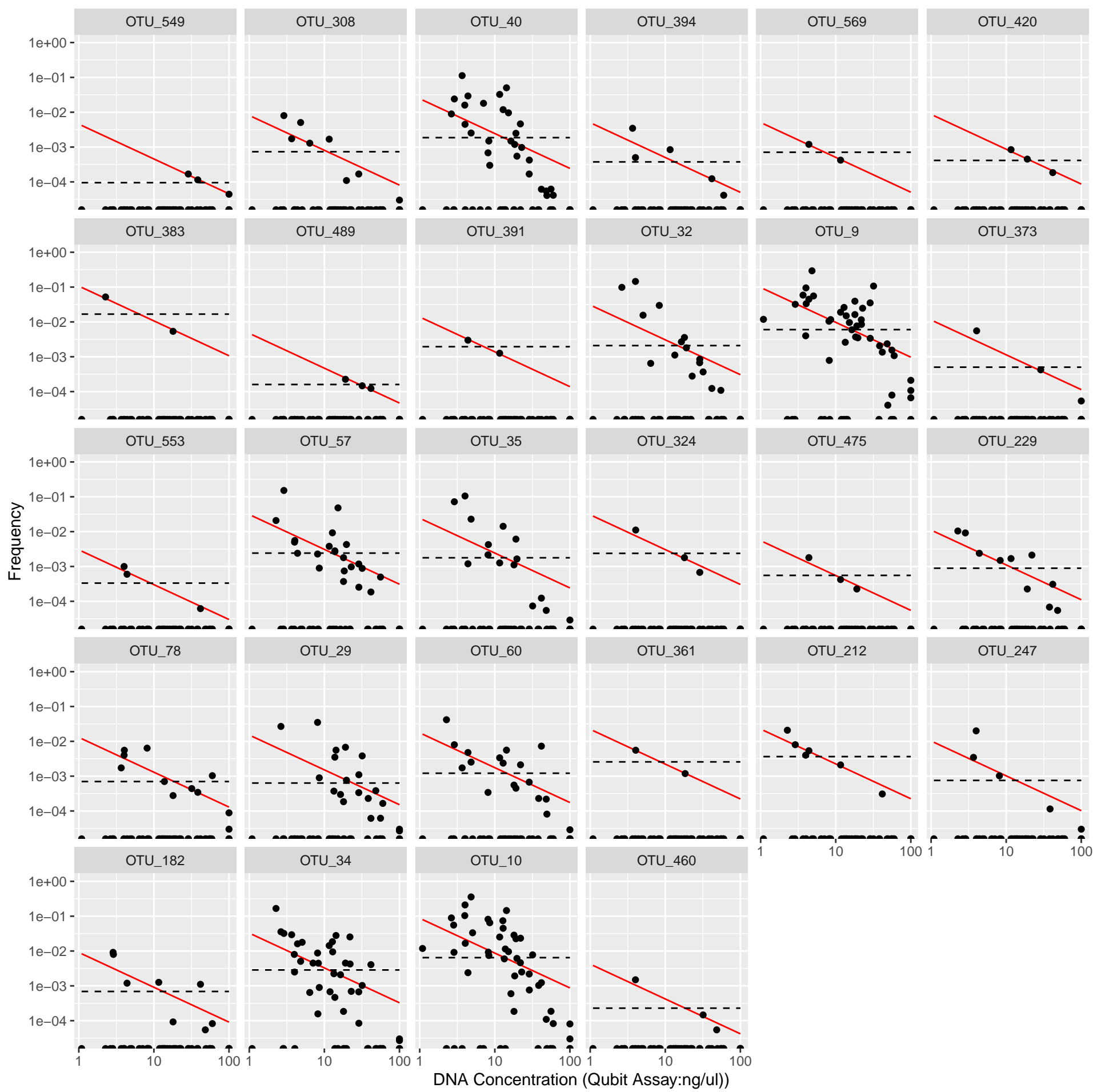

Supplement: S1 Fig — The frequencies of OTUs were inspected as a function of DNA concentration (range: 0.087 to 100 ng/μL) measured by Qubit assay after 16S V4 PCR and prior to Miseq sequencing. An OTU is classified as contaminant or non-contaminant by comparing its associated score statistic P (decontam score) to the default classification threshold of 0.1. A total of 28 OTUs were identified as contaminants using the frequency based decontam classification at the default threshold. The frequency of all contaminant OTUs showed negative correlation with the total DNA concentration (red line). The horizontal black dot line represents the expected frequency of non-contaminant OTUs. (PDF) [file pone.0227967.s001.pdf]

○ Uninfected ● Infected

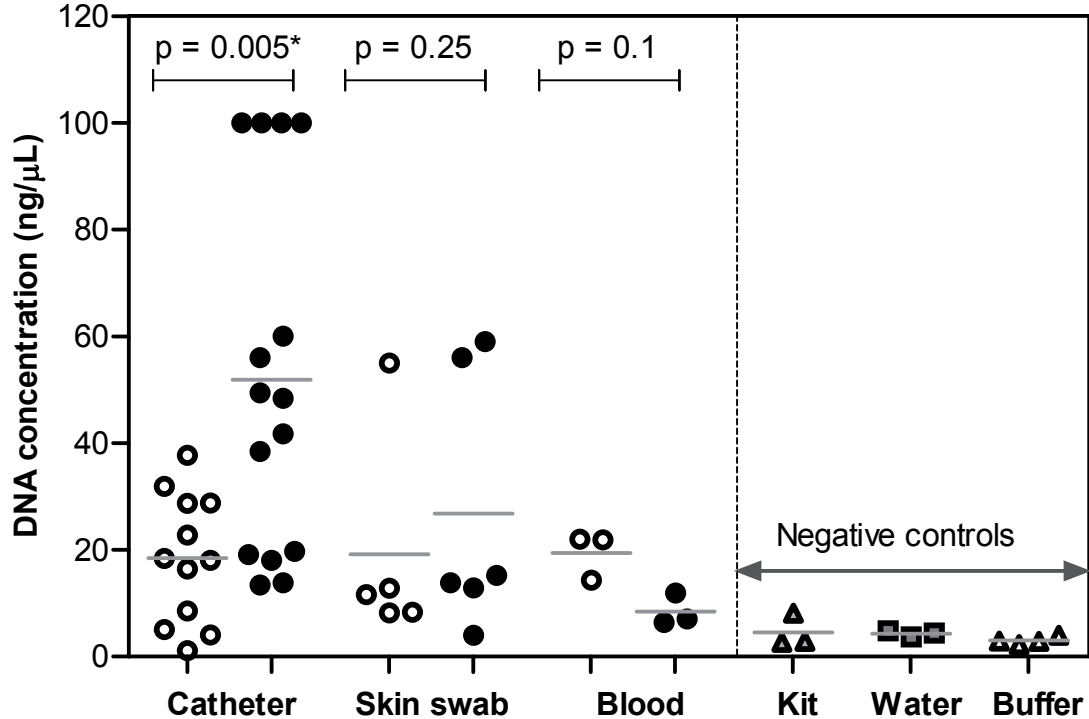

Supplement: S2 Fig — Significantly higher amount of the PCR amplicon was detected in the infected catheters (n = 15) compared to the uninfected catheters (n = 12) (Mann-Whitney test p<0.05). There was no significant difference in the amount of 16S V4 PCR amplicon between infected and uninfected skin swabs (p>0.05) as well as between the skin samples and the negative controls (Kruskal Wallis with Dunn’s post-hoc test p>0.05). (PDF) [file pone.0227967.s002.pdf]

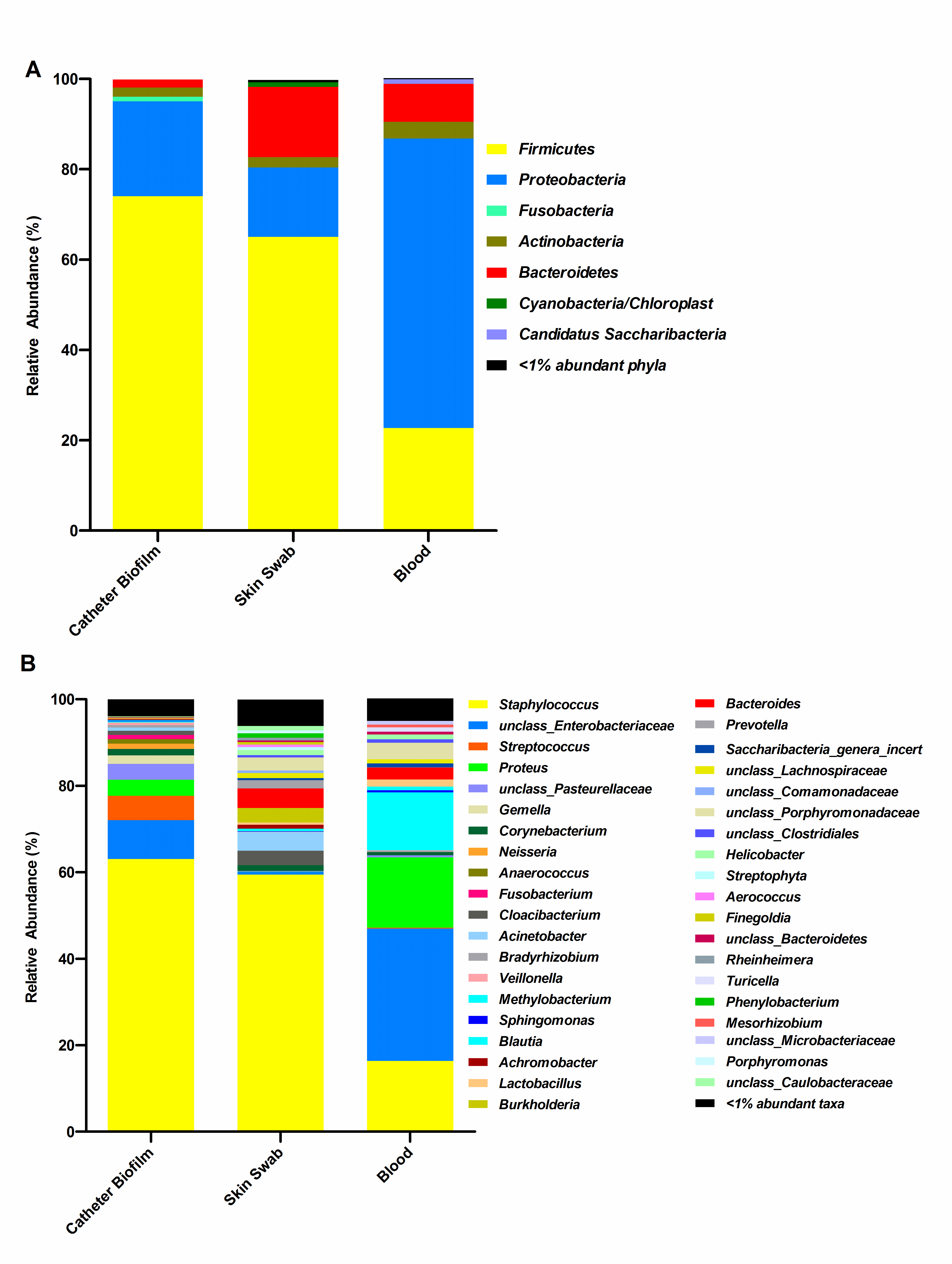

Supplement: S4 Fig — Bar plots show (A) phylum and (B) genus (wherever possible) level bacterial taxa present in the catheter biofilm (n = 27), skin swab (n = 11) and blood (n = 6) samples of the uninfected and infected neonates combined together. Taxa at ≥1% relative abundance in at least one of the samples in each group are reported individually, while those with <1% abundance are grouped together under ‘<1% abundant’ category. (TIF) [file pone.0227967.s004.tif]
