# Supplementary material for: Microbiome signatures in neonatal central line associated bloodstream infections
Source: PLoS One. 2020 Jan 16;15(1):e0227967. doi: 10.1371/journal.pone.0227967 (PMC6964844; doi:10.1371/journal.pone.0227967)
Supplement: S1 Table — (DOCX) [file pone.0227967.s006.docx]

**S1 Table. Clinical information of the patients in the study**

| S.N. | Bwt (g) | GA (wk) | Catheter days | NEC | Blood culture | Maximum feeds (ml/kg/day) | Predominant nutrition  (> 50%) | CLABSI |
| --- | --- | --- | --- | --- | --- | --- | --- | --- |
| 1 | 700 | 24 1/7 | 16 | No | No | 130 | MEBM | No |
| 2 | 2210 | 35 3/7 | 65 | No | No | 10 | TPN | No |
| 3 | 1800 | 32 3/7 | 15 | No | No | 140 | MEBM | No |
| 4 | 3400 | 40 3/7 | 11 | No | No | 150 | MEBM | No |
| 5 | 700 | 24 1/7 | 28 | No | No | 120 | TPN | No |
| 6 | 570 | 25 5/7 | 22 | No | No | 130 | TPN | No |
| 7 | 941 | 26 3/7 | 23 | No | No | 140 | MEBM | No |
| 8 | 3285 | 39 5/7 | 11 | No | No | 110 | TPN | No |
| 9 | 1009 | 28 | 6 | No | No | 100 | DEBM | No |
| 10 | 1545 | 29 5/7 | 6 | No | No | 120 | MEBM | No |
| 11 | 1445 | 29 5/7 | 5 | No | No | 120 | MEBM | No |
| 12 | 2625 | 34 3/7 | 18 | No | No | 150 | DEBM | No |
| 13 | 1215 | 29 3/7 | 10 | No | No | 150 | MEBM | No |
| 14 | 3060 | 39 | 12 | No | No | 120 | TPN | No |
| 15 | 2110 | 34 1/7 | 50 | Yes | No | 140 | TPN | No |
| 16 | 652 | 24 3/7 | 49 | Yes | *S. aureus* | 60 | TPN | Yes |
| 17 | 641 | 26 3/7 | 9 | Yes | *Candida spp*. and  *S. epidermidis* | 0 | TPN | Yes |
| 18 | 3360 | 39 | 12 | No | CONS | 60 | TPN | Yes |
| 19 | 1295 | 26 4/7 | 49 | No | *Bacillus spp*. and *Enterobacter spp*. | 20 | TPN | Yes |
| 20 | 2305 | 37 1/7 | 6 | No | *E. coli* | 0 | TPN | Yes |
| 21 | 655 | 25 3/7 | 17 | Yes | CONS, *S. aureus* | 90 | TPN | Yes |
| 22 | 740 | 26 | 13 | No | CONS, *Enterococcus faecalis* | 120 | TPN | Yes |
| 23 | 2574 | 35 3/7 | 73 | No | *Enterobacter spp.* | 80 | TPN | Yes |
| 24 | 1349 | 31 | 4 | No | *S. aureus* | 40 | TPN | Yes |
| 25 | 595 | 24 1/7 | 6 | Yes | CONS, *Candida spp.* | 0 | TPN | Yes |
| 26 | 2829 | 35 | 46 | No | *Proteus mirabilis* | 20 | TPN | Yes |
| 27 | 835 | 24 4/7 | 8 | No | *S. epidermidis* | 80 | TPN | Yes |
| 28 | 890 | 28 6/7 | 26 | No | *S. epidermidis* | 10 | TPN | Yes |
| 29 | 2495 | 34 6/7 | 33 | Yes | *S. epidermidis* | 100 | TPN | Yes |
| 30 | 601 | 26 4/7 | 7 | No | CONS, *S. aureus* | 40 | TPN | Yes |

**Note**: Bwt, birth weight; GA, gestational age; NEC, necrotizing enterocolitis; CLABSI, catheter-associated bloodstream infections; TPN, Total parenteral nutrition; MEBM, Mother’s expressed breast milk; DEBM, Donor expressed breast milk; CONS, Coagulase Negative Staphylococci.
