# Supplementary material for: Microbiome signatures in neonatal central line associated bloodstream infections
Source: PLoS One. 2020 Jan 16;15(1):e0227967. doi: 10.1371/journal.pone.0227967 (PMC6964844; doi:10.1371/journal.pone.0227967)
Supplement: S2 Table — (DOCX) [file pone.0227967.s007.docx]

**S2 Table. Number of reads in *decontam*-identified contaminant OTUs**

| OTU_ID | Total reads | Taxonomy |
| --- | --- | --- |
| OTU_9 | 2585 | *Root; k__Bacteria; p__Actinobacteria; c__Actinobacteria; o__Actinomycetales; f__Propionibacteriaceae; g__Propionibacterium* |
| OTU_10 | 910 | *Root; k__Bacteria; p__Proteobacteria; c__Gammaproteobacteria; o__Pseudomonadales; f__Moraxellaceae; g__Acinetobacter* |
| OTU_34 | 215 | *Root; k__Bacteria; p__Bacteroidetes; c__Bacteroidia; o__Bacteroidales; f__Bacteroidaceae; g__Bacteroides* |
| OTU_40 | 151 | *Root; k__Bacteria; p__Bacteroidetes; c__Bacteroidia; o__Bacteroidales; f__Porphyromonadaceae; g__Odoribacter* |
| OTU_60 | 150 | *Root; k__Bacteria; p__Firmicutes; c__Clostridia; o__Clostridiales; f__Lachnospiraceae; g__Roseburia* |
| OTU_29 | 144 | *Root; k__Bacteria; p__Proteobacteria; c__Gammaproteobacteria; o__Pseudomonadales; f__Pseudomonadaceae; g__Pseudomonas* |
| OTU_57 | 121 | *Root; k__Bacteria; p__Proteobacteria; c__Gammaproteobacteria; o__Pseudomonadales; f__Moraxellaceae; g__Enhydrobacter* |
| OTU_35 | 95 | *Root; k__Bacteria; p__Proteobacteria; c__Gammaproteobacteria; o__Oceanospirillales; f__Halomonadaceae; g__Halomonas* |
| OTU_32 | 81 | *Root; k__Bacteria; p__Proteobacteria; c__Betaproteobacteria; o__Neisseriales; f__Neisseriaceae; g__unclassified_Neisseriaceae* |
| OTU_78 | 46 | *Root; k__Bacteria; p__Actinobacteria; c__Actinobacteria; o__Actinomycetales; f__Corynebacteriaceae; g__Corynebacterium* |
| OTU_182 | 25 | *Root; k__Bacteria; p__Firmicutes; c__Clostridia; o__Clostridiales; f__Lachnospiraceae; g__unclassified_Lachnospiraceae* |
| OTU_229 | 14 | *Root; k__Bacteria; p__Firmicutes; c__Negativicutes; o__Selenomonadales; f__Veillonellaceae; g__Dialister* |
| OTU_212 | 11 | *Root; k__Bacteria; p__Bacteroidetes; c__Bacteroidia; o__Bacteroidales; f__Porphyromonadaceae; g__Parabacteroides* |
| OTU_324 | 11 | *Root; k__Bacteria; p__Bacteroidetes; c__Sphingobacteriia; o__Sphingobacteriales; f__Sphingobacteriaceae; g__Sphingobacterium* |
| OTU_308 | 9 | *Root; k__Bacteria; p__Bacteroidetes; c__unclassified_Bacteroidetes; o__unclassified_Bacteroidetes; f__unclassified_Bacteroidetes; g__unclassified_Bacteroidetes* |
| OTU_361 | 9 | *Root; k__Bacteria; p__Bacteroidetes; c__Flavobacteriia; o__Flavobacteriales; f__Flavobacteriaceae; g__Flavobacterium* |
| OTU_373 | 8 | *Root; k__Bacteria; p__Bacteroidetes; c__Sphingobacteriia; o__Sphingobacteriales; f__Sphingobacteriaceae; g__unclassified_Sphingobacteriaceae* |
| OTU_247 | 7 | *Root; k__Bacteria; p__Proteobacteria; c__Alphaproteobacteria; o__Sphingomonadales; f__Sphingomonadaceae; g__Sphingomonas* |
| OTU_420 | 7 | *Root; k__Bacteria; p__Bacteroidetes; c__Bacteroidia; o__Bacteroidales; f__Rikenellaceae; g__Alistipes* |
| OTU_489 | 5 | *Root; k__Bacteria; p__Firmicutes; c__Clostridia; o__Clostridiales; f__Lachnospiraceae; g__unclassified_Lachnospiraceae* |
| OTU_394 | 5 | *Root; k__Bacteria; p__Firmicutes; c__Clostridia; o__Clostridiales; f__Lachnospiraceae; g__unclassified_Lachnospiraceae* |
| OTU_549 | 4 | *Root; k__Bacteria; p__Firmicutes; c__Bacilli; o__Bacillales; f__Staphylococcaceae; g__unclassified_Staphylococcaceae* |
| OTU_383 | 3 | *Root; k__Bacteria; p__Bacteroidetes; c__Flavobacteriia; o__Flavobacteriales; f__Flavobacteriaceae; g__Capnocytophaga* |
| OTU_391 | 3 | *Root; k__Bacteria; p__Firmicutes; c__Clostridia; o__Clostridiales; f__Lachnospiraceae; g__unclassified_Lachnospiraceae* |
| OTU_460 | 3 | *Root; k__Bacteria; p__Proteobacteria; c__Alphaproteobacteria; o__Rhodospirillales; f__Acetobacteraceae; g__Roseomonas* |
| OTU_475 | 2 | *Root; k__Bacteria; p__Proteobacteria; c__Betaproteobacteria; o__Burkholderiales; f__Sutterellaceae; g__Parasutterella* |
| OTU_569 | 1 | *Root; k__Bacteria; p__Firmicutes; c__Clostridia; o__Clostridiales; f__Lachnospiraceae; g__unclassified_Lachnospiraceae* |
| OTU_553 | 1 | *Root; k__Bacteria; p__Bacteroidetes; c__Bacteroidia; o__Bacteroidales; f__Rikenellaceae; g__Alistipes* |
