# Supplementary material for: Microbiome signatures in neonatal central line associated bloodstream infections
Source: PLoS One. 2020 Jan 16;15(1):e0227967. doi: 10.1371/journal.pone.0227967 (PMC6964844; doi:10.1371/journal.pone.0227967)
Supplement: S3 Table — (DOCX) [file pone.0227967.s008.docx]

**S3 Table. Differently abundant bacterial taxa in uninfected and infected catheter biofilms**

| S.N. | Taxa | Mean relative abundance (%) | | p-value (Mann-Whitney test) |
| --- | --- | --- | --- | --- |
|  |  | **Uninfected catheter**  **(n=12)** | **Infected catheter**  **(n=15)** |  |
|  | *g__Bradyrhizobium* | 1.18 | 0.13 | 0.001 |
|  | *g__**Cloacibacterium* | 1.72 | 0.32 | 0.005 |
|  | *g__Sphingomonas* | 0.36 | 0.04 | 0.009 |
|  | *g__Methylobacterium* | 0.001 | 0.43 | 0.009 |
|  | *g__Tumebacillus* | 0.39 | 0.16 | 0.009 |
|  | *g__Proteus* | 0.001 | 6.68 | 0.010 |
|  | *g__Faecalibacterium* | 0 | 0.08 | 0.017 |
|  | *g__Chryseobacterium* | 0.19 | 0 | 0.020 |
|  | *g__unclassified_Ruminococcaceae* | 0 | 0.04 | 0.034 |
|  | *g__unclassified_Staphylococcaceae* | 0 | 0.002 | 0.034 |
|  | *g_Lactococcus* | 0.01 | 0.00 | 0.049 |

Note: g=genus; n=number of samples.
