# Supplementary material for: Microbiome signatures in neonatal central line associated bloodstream infections
Source: PLoS One. 2020 Jan 16;15(1):e0227967. doi: 10.1371/journal.pone.0227967 (PMC6964844; doi:10.1371/journal.pone.0227967)
Supplement: S4 Table — (DOCX) [file pone.0227967.s009.docx]

**S4 Table. The effect of nutrition on catheter microbiota composition in neonates**

| S.N. | Taxa | Mean relative abundance (%) | | p-values (Mann-Whitney test) |
| --- | --- | --- | --- | --- |
|  |  | **TPN**  **(n=20)** | **Enteral feeds***  **(n=7)** |  |
|  | *g__unclassified_Ruminococcaceae* | 0.03 | 0 | 0.003 |
|  | *g__Flavonifractor* | 0.002 | 0 | 0.018 |
|  | *g__Proteus* | 5.01 | 0.003 | 0.018 |
|  | *g__Sphingopyxis* | 0.001 | 0 | 0.018 |
|  | *g__Nocardiopsis* | 0.001 | 0 | 0.018 |
|  | *g__Leuconostoc* | 0.002 | 0.02 | 0.022 |
|  | *g__unclassified_Clostridia* | 0.001 | 0 | 0.023 |
|  | *g__Caulobacter* | 0.01 | 0 | 0.027 |
|  | *g__Paenibacillus* | 0 | 0.01 | 0.028 |
|  | *g__Nocardioides* | 0.004 | 0 | 0.037 |

Note: g=genus; n=number of samples; *Enteral feeds=MEBM and DEBM combined.
